# Medico-Chirurgical Transactions

**Published:** 1818-01

**Authors:** 


### *Medico-Chirurgical Transactions, Vol. VIII. Part I.*

( Continued from Vol. IV, page 496. )

Art. 6. *On the internal and external Use of the Nitro-muriatic Acid, in the cure of Diseases.* By H. Scott, M.D.

In the earlier part of his practice in India, Dr. Scott entertained a high opinion of the oxydes of mercury in the treatment of those modifications of disease which occur in that climate; but, on more ample experience, he became "frequently much dissatisfied with their effects."

The acid first used by the Doctor as a substitute for mercury, was procured from impure Bengal saltpetre, by means of alum; and was in reality, a mixture of the nitric and muriatic acids. Attributing the good effects produced to the former only, that acid was procured of considerable purity; but its operation was "less decisive, less valuable, and of a different kind."

The disagreeable effects sometimes produced on the teeth and stomach by the internal exhibition of this acid, suggested its external use in the form of a bath.—A quantity of warm water being rendered more or less acidulous, according to the irritability of the patient's skin, by the addition of a proportionate quantity of acid. In such a bath, the Doctor, who laboured under some hepatic affection, by way of experiment, immersed himself to the chin for half an hour, five following days. After the second bathing, he had "an odd sensation about the gums, jaws, and teeth." After the third, uneasiness in swallowing, reddened gums, and disposition to salivation." After the fourth, a sense of burning over the palate and down the gullet, like what arises from chewing an acrid vegetable. After the fifth, the mouth somewhat pained, but not ulcerated. Digestion improved; the liver, "unclogged by disease, is doing its office with facility." Quickened circulation and some affection of the mouth continued for a fortnight after discontinuing the bath.

Dr. Scott now prefers equal parts of the nitric and muriatic acids for external use; and in this country is satisfied with immersing the legs and feet only. He observes also, that sponging the body with the diluted acid produces the same effects with bathing.

With the exception of the ulcerated mouth and fœtid breath, the effect produced by the nitro-muriatic acid are very similar to those of mercury. They are, however, more fugitive;—the salivation may be excessive one hour, entirely disappear the next, and perhaps return the third.

"As a very general rule for its employment, it may be observed, that whenever the mercurial preparations are indicated, this acid will be found useful, with this difference, that in cases where mercury is highly injurious from peculiarity of constitution or other causes, the nitro-muriatic acid may be employed with safety and advantage." "On the other hand," continues the Doctor, "I should not at present recommend it in acute diseases, with the exception of some kinds of fevers, and of hepatitis of every character. Where the pulse is quick, and where there is an inflammatory tendency, I think it would be injurious."

In the purely acute hepatitis, the propriety of employing the acid is questionable, and must not supersede the use of mercury; but in that state of liver so frequent in warm climates, where both acute and chronic inflammation exist in different parts of that viscus at the same time, the medicine in question, applied to the skin, is the "most effectual, and the safest remedy." It must be remembered however, that

"—there is no security against relapse, till the health and strength are fully restored; and that, till then, some repetitions of the remedy are necessary."

The beneficial effects of the nitro-muriatic practice cannot be appreciated until some weeks or months from its disuse. Whilst under its influence, it is not uncommon for the patient to lose flesh, complain of increased uneasiness in the liver, to have the secretion of bile augmented, and the skin tinged yellow.

In the external use of this medicine, the sensibility possessed by the patient's skin must be attended to. "I have," says the Doctor, "immersed many to the chin in the bath, and I have been afraid, in other cases, to wet more than a single hand with the acid." Should considerable effects be produced, it is necessary to suspend the use of the acid for a week or two, as it may cause unnecessary uneasiness from "bilious discharges and bilious feelings."

We shall transcribe the following paragraph, leaving our readers to comment on it as they please.

"On what principle to account for the singular agency of this compound acid on the skin, I confess myself in great doubt, or rather *in total ignorance.* That this power depends on chlorine, an elementary substance according to Sir H. Davy, there cannot be a doubt. The almost instantaneous effects that it produces on some people, its operation on the stomach, which it stimulates occasionally to contraction, and its suddenly causing a flow of bile, are all unlike a remedy that is conveyed by the known channels of absorption. In my opinion, the taste and sensation that it occasions in the mouth, are exactly such as are produced by the galvanic fluid; and it would be presumptuous to affirm, that no agency of a similar kind has an influence in the effects of the nitro-muriatic acid. I can suppose that the effects of this remedy do not arise from the transfer of matter by any set of vessels; but that they are the consequence of peculiar motions, which it has the power of exciting in the solids, or the fluids of the body."

In a postscript to the communication, we are informed that sponging the body with a solution of chlorine (water saturated with oxymuriatic acid gas) has the same effects with the nitro-muriatic acid, and proves less irritating to the skin; and, that we are to attribute the sanative powers of the murias and oxymurias hydrargyri to the *chlorine* these preparations contain.

Art. 7. *History of a Case of ill-conditioned Ulcer of the Tongue successfully treated by Arsenic,* in a Letter from Charles Lane, Esq. to Henry Cline, Esq. President

G. B. aged 23, applied to Mr. Lane with a "foul ulcer beneath the tongue"; and said, that some time before, he had one on the upper part, which was now healed. There was a deep, irregular fissure, with raised, jagged, hardened edges upon the upper surface of the tongue, communicating with the ulcer beneath it. From this last mentioned ulcer, a probe passed through the substance of the tongue into a deep ulceration at the root of that organ, and thence into the throat. The disease had a carcinomatous aspect. General health much affected. Various plans of treatment were tried, without benefit. Mercury produced "evident bad effects." At last, arsenic was thought of. Ten minims (gradually increased to seventeen) of Fowler's solution were given every eight hours, and the ulcer injected twice a day with a lotion containing some of the same. At the end of a month, it was perfectly healed; but from irregularity in living, soon re-appeared, and yielded in three weeks to the same treatment. From the same cause, the ulcer returned a third time, more aggravated, and accompanied by all "its horrid symptoms." At the suggestion of Mr. Cline, a drachm of pulvis sarsæ was given twice a day in addition to the arsenic solution, which, to prevent relapse, was continued for ten days after the healing of the ulcers. There has been no return of the complaint from that time, which is now three years since.

Art. 8. *History of a Case of Lithotomy, with a few Remarks on the best Mode of making the Incision in the lateral Operation.* By S. Cooper, Esq. Surgeon to the Forces.

Mr. Cooper commences his paper with observing, that in no operation of surgery do "mortifying failures and dangerous blunders" so often occur, as in that of lithotomy. Our own observation bears testimony to the truth of this remark.

The purport of the present communication is

"—to recite a case of lithotomy attended with some particularity, and then offer a few general observations on the proposed direction and size of the incision."

The case alluded to, is that of a boy, 4 years of age, on whom Mr. C. performed the operation of lithotomy, whilst in Holland, in 1814; a scalpel, and a small silver catheter being the only instruments at hand.

"After making the external incision in the usual way," says Mr. C. "and dividing the membranous part of the urethra, I dissected along the side of the catheter, until the prostate gland, and a small portion of the bladder beyond it, could be plainly felt with the fore finger of my left hand. In this step of my operation, the edge of the knife was constantly directed inwards and upwards; the catheter was then withdrawn. The prostate gland now served as my guide in the completion of the internal part of the incision. With the edge of the knife, therefore, directed inwards and upwards, I cut into the bladder behind the base of the prostate gland; and carrying the incision forwards under the direction of my left fore finger, I made the requisite division of the neck of the bladder and upper part of the side of that gland. With a small pair of ordinary dressing forceps, a roundish stone, about an inch and a quarter in diameter, was readily extracted."

This case, which did extremely well, is related to impress on the minds of surgeons, the advantages attending a free incision of the parts in lithotomy. The incision in the integuments, continues Mr. Cooper,

"—should be at least three or four inches in length, and directed towards a point situated a very little towards the anus, from the innermost projection of the tuberosity of the ischium. From the line thus made, the incision should be carried inwards and upwards through all the parts between it and the side of the prostate gland."

Thus the axis of the wound will be as straight and direct as possible; by which the extraction of the stone is facilitated, and the pubic artery, rectum, and seminal ducts escape unhurt.

Scarpa seems averse to making a free incision of the prostate gland and bladder, fearing that effusion of urine, suppuration, gangrene, and fistula, would be the consequence; but we certainly agree with Mr. Cooper in asserting, that these evils are more likely to occur where the opening into the bladder is narrow and confined.

The adoption of the scalpel or beaked knife, and disuse of the gorget at Bartholomew's, Mr. Cooper considers as a beneficial change of practice. The latter instrument, he observes, in general, made too small an opening into the bladder; and, as in such a case, violence was often used in extracting the calculus with the forceps, the occurrence of peritonæeal inflammation, that insidious enemy which proves so destructive to patients after lithotomy, was rendered more frequent.

Art. 9. *Case of fatal Hæmorrhage from the Extraction of a Tooth.* By Richard Blagden, Esq. Surgeon Extraordinary to the Duke of Kent.

The subject of this case, while a boy, had an alarming hæmorrhage from the alveolus follow the extraction of a tooth, and continue twenty-one days. After this a slight wound was always attended with bleeding, difficult to stop. In his twenty-sixth year, a trifling wound on the forehead, which divided an artery, gave origin to a profuse hæmorrhage, which neither pressure, styptics, ligatures, nor any thing but the application of kali purum suppressed.

The second dens molaris of the upper jaw on the left side became carious; the fear of hæmorrhage caused the patient to defer its extraction for some time, but the pain it occasioned at last induced him to submit to the operation. A profuse hæmorrhage followed. Mr. Blagden being called in the next morning, applied lunar caustic, and that failing, stopped the socket with sponge soaked in a solution of blue vitriol, directing cold applications to the face: this procured only a temporary suspension of the hæmorrhage. On the fourth day Mr. Brodie applied the cautery, which restrained the bleeding six hours. The alveolus was again stopped with sponge, and the cauterization repeated twice, without avail. On the fifth day Mr. Brodie tied the left common carotid, but this measure had no beneficial effect. There was a profuse oozing (which ceased only during the application of ice, returning immediately on its removal) from the surface of the wound made in the operation. Ice was also applied to the left side of the face, and the hæmorrhage was suspended for a few hours. It afterwards recurred, and the patient died the next morning; being a week from the removal of the tooth.

The carotid was found of its natural texture, except having several opaque spots on the outer surface of its inner coat, such as precede ossification. The coats of the temporal and other branches were so attenuated as to be nearly transparent.

ART. 10. *Rupture of the Stomach and Escape of its Contents into the Cavity of the Abdomen.* By JOHN CRAMPTON, M.D. King's Professor of Materia Medica, and Assistant Physician to Steeven's Hospital, Dublin. Communicated by Dr. BAILLIE, with additional Observations by B. TRAVERS, Esq. F.R.S. Vice President.

A young lady, aged 29, of spare habit and sallow complexion, subject to pain and hypochondria, was seized with agonizing pains in the abdomen, extending to the back and shoulders. Abdominal muscles contracted; belly hard, but not tumid. No acceleration of pulse; foulness of tongue, or nausea. The pain continued without a moment's respite, notwithstanding venæsection, fomentations, blisters, enemas, the warm bath, and opiates. Her extremities became cold, pulse indistinct, and she gradually sunk, expiring in agony early the next morning.

*Sectio Cadaveris, thirty-six hours after Death.* Stomach pale, flaccid, and empty; its contents having escaped into the abdominal cavity through a round aperture on its anterior surface, at the junction of the cardiac and pyloric portions. This opening was about the size of a pea, and appeared the result of an ulcer on the mucous surface, which had gradually penetrated the other coats. Peritonæum inflamed; intestines adhering to each other by exudations of lymph. Liver and spleen shrunk, not indurated.

The following Cases and Observations are added by Mr. Travers.

*Case* 1. A man, aged 35, of a strumous habit, but healthy, while dining, was seized with excruciating pain in the abdomen, particularly at the umbilical region, shooting from thence to the neck and shoulders. Abdomen tense and hard; respiration agitated; pulse unaffected. Expulsion of flatus from the stomach, but no disposition to vomit. The symptoms increased in violence, the extremities became cold, the pulse indistinct, and he died thirteen hours after the attack.

*Examination.* Peritonæum inflamed; folds of intestine adhering to each other; a quantity of bile in the pelvis. There was a circular foramen in the stomach admitting a writing pen, a finger's breadth below the pylorus. It was the centre of an ulcer on the mucous coat, which included two-thirds of the ring of the pylorus.

*Case* 2. A bon vivant, aged 30, was seized with pain in the abdomen while singing a song. He lived for two or three days in great suffering. Coagulable lymph with alimentary matter were effused into the peritonæal cavity. The latter issued from an ulcerated aperture in the small intestines.

*Case* 3. A hair-dresser, subject to violent abdominal pain, which was always relieved by brandy, experienced a most violent attack, which terminated his existence in thirty-six hours. There was a circular aperture at the union of the duodenum and stomach, being the centre of an ulceration half an inch in extent, which had destroyed the villous and muscular coats of the bowel. Coagulable lymph was effused about the pylorus, but no adhesion had taken place.

From the sudden manner in which the violent symptoms came on after taking food, and the appearance of the aperture in the stomach, Mr. Travers concludes, that, in the preceding cases, the peritonæal coat was ruptured. Nature generally provides against effusion, by producing adhesions of contiguous surfaces. An ulcer of the colon was stopped by a piece of omentum; an ulcer of the stomach by adhesion to the root of the diaphragm. Effusion was prevented, in a third instance, by adhesion to the pancreas.

"What, it seems natural to inquire, are the circumstances which determine this important difference of result?—When previous disease exists, it is well known, that the preservative power is less readily excited than when the general health is good. But the health of the unhappy persons, the subjects of these cases, was not obviously impaired; and I imagine, therefore, that a mechanical lesion, or some other local circumstance, must influence the event."

The following case seems to prove, it is influenced not by constitutional but local causes, and that "it is the size and destructiveness of the ulcer of the mucous coat which excites the adhesive inflammation of the peritonæum."

An aged woman had symptoms of strangulated hernia. A small femoral hernia in the right groin was easily reduced, but the symptoms continued till her death. An aperture, the size of a pea at its pyloric extremity, gave exit to the fæculent contents of the stomach, which were found in the cavity of the abdomen. The mucous coat of the stomach was highly vascular. Near its larger curvature, on the posterior surface, a large ulcer, with thickened edges, firmly adhered to the anterior surface of the pancreas.

The paper concludes with the following diagnostics of this irremediable affliction:

"1. Sudden, most acute, and unremitting pain, radiating from the scrobiculus cordis to the circumference of the trunk, and even to the limbs. I may add a peculiar pain, though I know not how to describe the peculiarity: its intensity, like that of parturition, absorbs the whole mind of the patient, who, within an hour from the enjoyment of perfect health, expresses his serious and decided conviction, that if the pain be not speedily alleviated, he must die.

"2. Coeval with the attack of pain, remarkable rigidity and hardness of the belly, from a fixed and spastic contraction of the abdominal muscles.

"3. A natural pulse for some hours, until the symptoms are merged in those of acute peritonitis, and its fatal termination in the adhesive stage."

Art. 11. *An Account of a Case, where a severe nervous Affection came on after a punctured Wound of the Finger, and in which Amputation was successfully performed.* By J. Wardrop, Esq. F. R. S. Ed.

A woman, aged 48, a year prior to her application to Mr. W. pricked the fore finger of the right hand, near the point, with a gooseberry thorn. Pain and inflammation followed, extending in a few days all along that finger and the adjoining phalanx of the next. After three months, the pain and swelling, except in the two first phalanges of the wounded finger, disappeared. Disorder of the general health, with nervous paroxysms, succeeded. The point of the finger became excessively painful, the skin being so acutely sensible, that the patient "could not hold a pin betwixt the finger and thumb to save her life." Except a slight red spot on the point, no change could be perceived in the finger. The nervous paroxysms generally occurred twice or thrice in the day; one always came on when rising in a morning.

"During these attacks, the pain extended along the finger to the back of the hand, and between the two bones of the fore-arm; darted through the elbow joint, stretched up the back of the arm to the neck and head, producing a sensation at the root of the hairs, as if they had become erect. To these feelings succeeded a dimness of sight, and the pain afterwards went suddenly into the stomach, followed by sickness and vomiting. She had constantly the feeling of a lump in her stomach, and always vomited after taking food. Her flesh was much wasted, and she was extremely feeble."

Cooling lotions were used, and three incisions made in the point of the finger, without benefit. Amputation at the second joint quickly relieved, and permanently removed, every unpleasant symptom.

"On carefully dissecting the finger, no change could be detected in the structure of the nerves."

A few observations follow; and the author is of opinion, that in such cases as the present—

"It is preferable to sacrifice the limb, than attempt to save the patient's life by a simple division of the injured nerve."

Art. 12. *An Account of some remarkable Symptoms which were connected with a painful Affection of the Extremity of the left Thumb, together with the Mode of Treatment.* By John Pearson, Esq. F.R.S. Senior Surgeon to the Lock Hospital.

This is the case of a young lady, aged 18. The painful affection commenced suddenly, without assignable cause, near the extremity of the left thumb, and from thence extended to other parts, reducing her, in the space of ten months, to the following most distressing condition.

"The left arm considerably wasted, the thumb and fore finger so exquisitely sensible, that they could not endure the pressure of the slightest substance: the three contiguous fingers were inflected, firmly, into the palm of the hand; the elbow joint was contracted, and the whole arm painfully sensible. The right arm had acquired a high degree of sensibility, and was extremely weak; but there existed no contraction of any joint, nor conspicuous atrophy of the limb. She had extraordinary accessions of pain and spasm, during several hours, every two or three days, and on these occasions the muscles of the forehead and face were much disturbed by convulsive motions. Her health was delicate, but not sensibly impaired; the catamenia regular, but rather too abundant, and sometimes approaching to mænorrhagia. She suffered also from leucorrhœa and confined bowels."

Various remedies had been tried by the practitioners under whose care the young lady had been placed previous to her application to Mr. Pearson, without in the least benefiting, or even retarding, the progress of the complaint.

Having met with several untractable cases of local affection of the nerves, attended by muscular spasms, Mr. Pearson attempted their cure by "inflicting a disease which would extend over a large portion of the surface of the body," causing an extensive eruption, "attended by the usual concomitants of certain exanthemata." In the present case the following stimulating liniment was directed to be rubbed for ten minutes, twice a day, over the upper part of the arm:

℞. Olei olivæ ℥ijß; olei terebinthinæ ℥jß; acidi sulphurici ʒjß. Misce.

As this produced no effect, after three days the quantity of acid was increased half a drachm. The part soon became red, painful, and tumefied. Contrary to directions, the young lady persisted in applying the liniment after these local affections appeared, and the consequence was, that the whole limb, in little more than an hour, became more painful and tumid; and in five days, the arm and face were covered with a vesicular eruption, and the whole body partook of the tumefaction.

On the 5th day after the appearance of the eruption, a spontaneous motion of the thumb was observed, and she could touch the part without pain.

Our limits will not permit us to notice the minutiæ of the case farther; suffice it to say, the affected parts progressively lost their morbid sensibility, and the young lady was restored to perfect health.

A few observations on injuries and morbid alterations of the structure of the nerves are subjoined. The communication is interesting, and deserving of perusal.

ART. 13. *Cases of Fungus Hæmatodes, with Observations.* By G. LANGSTAFF, Esq. *And an Appendix, containing Two Cases of analogous Affections.* By W. LAWRENCE, Esq. F. R. S. &c.

*Case of Fungus Hæmatodes.*

In this instance a tumour, the size of a marble, appeared on the anterior part of the right leg, unattended with pain; and in four months acquired a diameter of two inches. Continuing to increase, the integuments were absorbed, and the fungus growth gradually protruded, discharging blood and ichor. Extirpation proved ineffectual, and amputation was performed above knee. The disease did not re-appear in the stump; but the man sunk, and died eight weeks after the operation.

On examining the limb, the fungus was found to originate from the cellular substance of the tibialis anticus and extensor longus, and consisted of "organized lymph, broken down coagulum, and a brain-like substance." Several tubera, of a similar substance, were found in the liver, lungs, and bronchial glands.

*Fungus Hæmatodes in the Urinary Bladder, Liver, and Lungs.*

This occurred in a pauper of 68 years.—*Symptoms.* Excruciating pain in the kidnies, bladder, and rectum. Constant desire to make water, which came away guttatim, and bloody. Dry cough; dyspnœa. The prostate gland was enlarged, and none but the smallest bougie could be got into the bladder. He died of symptoms resembling typhus.

*Dissection.* Bloody urine escaped through a rupture in the right ureter, and effused beneath the peritonæum. A fungus, the size of an orange, originating from the prostate gland, was found in the bladder, stopping up both the orifices of the ureters. The structure similar to the last case. Appearances of the disease in the liver and lungs.

### *Fungus Hæmatodes in the Lungs, Uterus, &c.*

Mrs. B. ætat. 52, mother of several children, was afflicted with a tumour in the abdomen, causing an enlargement equal to that of the last month of pregnancy. As it inclined to the left side, it was supposed to be an ovarian enlargement. Menstruation ceased four years prior to its appearance. Pain in the loins and turbid urine accompanied its growth. For the last year, pain in the chest, dyspnœa, emaciation, and hectic fever. She died like one suffocated.

*Examination.* A tumour, the size and figure of a large cocoa-nut, was placed rather obliquely across the spine, reaching from the concavity of the left ilium to the under surface of the liver. The omentum was stretched over it; the transverse arch of the colon made a deep furrow on its anterior and middle parts; and the descending arch, segmoid flexure, together with the small intestines, were greatly displaced. The cyst, containing the diseased growth, was loosely connected to the peritonæum by cellular substance; its veins were filled with firm coagula, and its contents were, exteriorly, irregular portions of greyish pulp, and softer parts mixed with coagulum; interiorly, a solid gelatinous substance like grated beef. Appearances of the disease were detected in the uterus; and in the spermatic veins, calculi, some of the size of a garden pea, were found, buried in the centre of round coagula of blood.

### *Fungus Hæmatodes of the Liver.*

A dram drinker, aged 66, was attacked with vomiting of blood and pain in the right hypochondrium. The liver could be felt greatly enlarged and painful. Conjunctivæ not much discoloured; fæces unhealthy, often black; urine deeply tinged with bile. His complaint was treated as hepatic, and for a time with some relief. Ascites, coma, and vomiting of blood, put a period to his sufferings. —*Examination.* Two gallons of serum in the abdomen. The liver enlarged, irregular, and studded with various sized tubercles. The covering of the convex surface of the large lobe cartilaginous, that of the left coated with lymph, through which some of the tubera protruded, presenting a true fungous appearance. In the liver were different sized effusions of coagulum and brain-like substance, without capsules, but retained by septa of organized lymph: most of its blood-vessels were filled with the same. Gall-duct obliterated; a large coagulum in the stomach; no abrasion, but the mucous coat very vascular. Large intestines filled with an inodorous, black, thick, mucous fluid, adhesive, tinging the fingers.

### *Fungus Hæmatodes in the Liver.*

A gentleman, aged 59, was afflicted, upwards of four years, with an hepatic affection; and for the last six months, with diarrhœa and intestinal hæmorrhage. He died icterical and emaciated.

*Examination.* A gallon of fluid in the abdomen. The liver not much enlarged, but presenting the same appearances, exteriorly and interiorly, as in the last case.

### *Fungus Hæmatodes in the Kidney.*

A man, aged 70, hemiplegic six years, had pain in the region of the left kidney, uneasiness in the rectum, difficulty in voiding, and (latterly) retention of urine. He died of apoplexy.

*Examination.* Effusion of serum between the cerebral membranes; vessels of the brain turgid, and recent or old sanguineous effusions in its substance; lateral ventricles distended with bloody serum; basilary and carotid artery partly ossified. Left kidney enlarged, its upper half presenting the appearance of fungus hæmatodes; the bladder of enormous capacity, muscular coats thickened, with hernia of the mucous one; prostate gland enlarged, its middle lobe acting as a valve, and impeding the discharge of urine.

### *Fungus Hæmatodes in the Kidney.*

Miss J. aged 21, had menstruated regularly from her 15th year. A week before a returning menstrual period, was suddenly attacked with vehement desire to make water, passing much limpid urine and florid blood. As considerable uterine irritation attended these symptoms, it was treated as a case of uterine hæmorrhage, and their violence abated. Retention of urine next appeared, and the catheter brought away large quantities of bloody fluid at each introduction.

When the period of menstruation expired, the irritation became less, the urine ceased to be bloody, but deposited a muco-purulent sediment, and her health somewhat improved. Pain in the right side was now complained of, and a pulsating tumour could be felt in the hypochondrium, projecting beyond the costal cartilages. This was attributed to hepatic disease, and treated with alterative doses of mercury; but it continued to increase in bulk, and the right thigh and leg became numbed. Bilious vomitings often occurred. Frequent hæmorrhages from the bladder; and the irritation of the disease, gradually brought the afflicted patient to the grave.

*Examination.* The inferior part of the diseased mass forming the tumour, and which consisted principally of the right kidney, rested on the concavity of the ilium, whilst its superior pressed against the under surface of the large lobe of the liver, compressing the gall-bladder and ducts. There were several purple spots on the peritonæum, omentum, and mesenteric glands, but no change of structure. Contents of the thorax and pelvis healthy. The right kidney, with a portion of adhering liver, weighed 11lb. 13oz.; the left 12oz. On cutting into the tumour, its cyst, which was the thickened capsula renalis, contained concentric coagula, and two pints of half dissolved blood mixed with pus; its internal surface was flocculent from attached coagulable lymph. In the left, and remaining part of the right kidney, were several tubera. Coarse injection passed from the enlarged trunk of the renal artery into the cavity of the sac.

*Cases of analogous Affections.* By Mr. Lawrence.

Case 1. A tumour, the size and shape of an orange, was removed from the left axilla of a healthy man, aged 43. It was vascular, lobulated, of homogeneous texture, and yielded a pulpy matter on scraping. The wound healed, and the man was discharged.

About four months afterwards he returned to the hospital with another tumour in the same axilla; others, moveable and painless, on the inner part of the left thigh and haunch, back of the neck, and scalp, with shortness of breath and constitutional irritation. He died two months after admission.

*Examination.* The axillary tumour adhered firmly to the ribs: its surface was irregular; its interior lobulated, and consisting of medullary matter, coagula, and firm septa. Tubercles, of a similar structure, were found in the lungs, heart, and, in short, in nearly all the thoracic and abdominal viscera. The cranial contents were free from the disease.

Case 2. Here the disease originated in the testis; this part had an uniform, rather elastic surface, and was greatly enlarged. The cord was thickened, and a tumour could be felt within the abdominal muscles.—The notes of this case were mislaid. The subject was 22 years of age, and tumefaction of the abdomen, bilious vomiting, and peritonitis, were the symptoms which preceded his death.

On dissection, no vestige of the natural structure of the testis remained. The exterior coats of that gland gave the swelling the elastic firmness it possessed. It consisted of cellular septa filled with matter similar to what has been so often described in the foregoing cases. Numerous tubercles of the disease were found in the omentum, and adhering about the pelvis, intermixed with recently effused coagula. A mass of soft matter, the bulk of a man's head, lay on the spine, behind the aorta and vena cava. This last vessel was closed for some extent. The spermatic vessels could not be found.

The liver and lungs also exhibited marks of the disease.

